# Multiple Functions of MiRNAs in *Brassica napus* L.

**DOI:** 10.3390/life12111811

**Published:** 2022-11-07

**Authors:** Jian Li, Yangyang Li, Rongyuan Wang, Jiangyan Fu, Xinxing Zhou, Yujie Fang, Youping Wang, Yaju Liu

**Affiliations:** 1 Xuzhou Institute of Agricultural Sciences in Jiangsu Xuhuai District, Xuzhou 221121, China; 2 Key Laboratory of Plant Functional Genomics of the Ministry of Education/Jiangsu Key Laboratory of Crop Genomics and Molecular Breeding, Yangzhou University, Yangzhou 225009, China

**Keywords:** miRNA, *Brassica napus*, development regulation, biotic stress, abiotic stress, transcriptome

## Abstract

The worldwide climate changes every year due to global warming, waterlogging, drought, salinity, pests, and pathogens, impedes crop productivity. *Brassica napus* is one of the most important oil crops in the world, and rapeseed oil is considered one of the most health-beneficial edible vegetable oils. Recently, miRNAs have been found and confirmed to control the expression of targets under disruptive environmental conditions. The mechanism is through the formation of the silencing complex that mediates post-transcriptional gene silencing, which pairs the target mRNA and target cleavage and/or translation inhibition. However, the functional role of miRNAs and targets in *B. napus* is still not clarified. This review focuses on the current knowledge of miRNAs concerning development regulation and biotic and abiotic stress responses in *B. napus*. Moreover, more strategies for miRNA manipulation in plants are discussed, along with future perspectives, and the enormous amount of transcriptome data available provides cues for miRNA functions in *B. napus*. Finally, the construction of the miRNA regulatory network can lead to the significant development of climate change-tolerant *B. napus* through miRNA manipulation.

## 1. Introduction

Amphidiploid *Brassica napus* L. (*B. napus* L.) is the third most popular oilseed crop after soybean and palm. It is widely planted and distributed in the world, and plays a vital role in vegetable oil, biofuel, and livestock feeding [1]. Nowadays, the rapeseed planting area of China ranks first in the world, but the total rapeseed production is still unable to meet the market demand with the increasing population and disruptive environmental conditions [2,3]. Biotic stresses (such as microbial infections) and abiotic stresses (such as drought, heat, flooding, salinity, etc.) are frequent and disruptive environmental conditions, creating various stresses that reduce the growth of biomass and the root system, leaf number, specific leaf area, photosynthesis, and chlorophyll content in *B. napus*, while stresses at the flowering or silique stage may lead to earlier flowering time and lower seed weight, oil content and fatty acid content, which greatly restrict the growth and development of rapeseed, and ultimately affect the yield and quality of rapeseed, and endanger food safety worldwide [4]. Therefore, improving the yield of rapeseed and deciphering the mechanism of rapeseed against various stresses are the most important strategies to meet the increasing edible oil demand [5,6].

**Figure 1:**
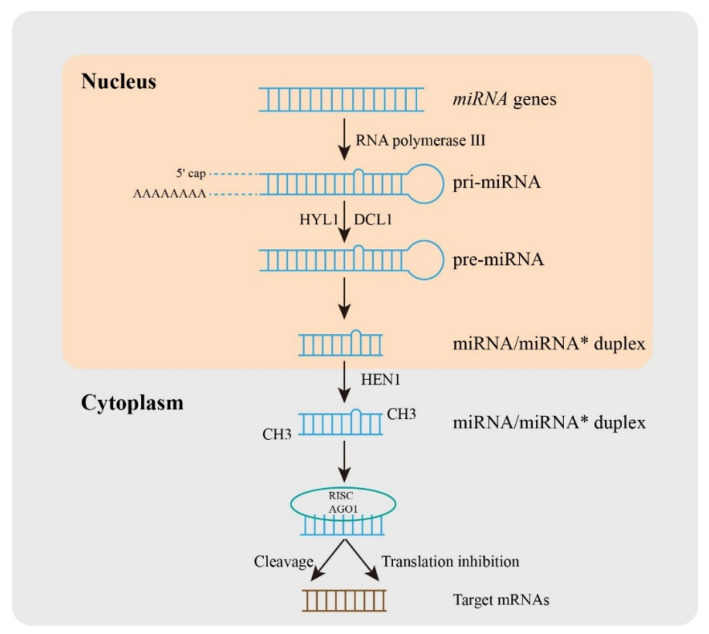
The processes of miRNA biogenesis in plants. In brief, a *miRNA* gene is transcribed into primary miRNA (pri-miRNA) with the help of RNA polymerase III, and then pri-miRNA is produced by the cleavage complex DICER like1 (DCL1), HYPONASTIC LEAVES1 (HYL1), and other proteins. The pre-miRNA is cleaved into double-stranded miRNA/miRNA*. The transporter carries it from the nucleus to the cytoplasm, and its 3′ end is methylated under the action of methyltransferase HUA-ENHANCER1 (HEN1), which eventually forms the double-stranded stable mature miRNA/miRNA*. Mature miRNAs are then loaded into RNA-induced silencing complex (RISC) and regulate gene expression.

Small RNAs (sRNAs) are 20–30 nucleotide long sections of non-coding RNA, including small interfering RNAs (siRNAs) [7], Piwi-interacting RNAs (piRNAs) [8], and microRNAs (miRNAs) [9]. The single-stranded miRNAs, acting as key regulators, are known to control the expression of target mRNAs and participate in the regulation of normal plant growth [10], development [11], as well as biotic and abiotic stress responses [12]. The first non-coding RNA was found in *Caenorhabditis elegans* in 1993 [13], and then Thomas Tuschl, David Bartel, and Victor Ambros used miRNA to name these small RNAs in published articles in 2001 [14]. Over the decades, emerging research on miRNA identification and characterization has given a new method for plant species improvement. More recently, Sanger developed the miRBase (http://www.mirbase.org/ URL (accessed on 7 October 2022)) database and established naming rules and usage specifications for miRNAs [15]. Then, a comprehensive and detailed database of small RNAs was built for plants, including miRFANs [16], TarDB [17], or sRNAanno [18]. In plants (Figure 1), the miRNA biosynthesis process includes the transcription of *miRNA* genes (*MIR*s) under the action of RNA polymerase III to produce the primary miRNA (pri-miRNA), and then the stem-loop structure is formed through 5′ caping and 3′ polyadenylation of long pri-miRNA in the nucleus [19]. The precursor miRNA (pre-miRNA) with a stem-loop structure is formed by the cleavage complex DICER like1 (DCL1) [20], HYPONASTIC LEAVES1 (HYL1) [21], and SERRATE (SE) [22], and then pre-miRNA is cut into double-stranded miRNA/miRNA* under the action of the cleavage complex [23]. The transporters carry it from the nucleus to the cytoplasm, and its 3′ end is methylated under the action of methyltransferase HUA-ENHANCER1 (HEN1), finally forming a double-stranded stable mature miRNA/miRNA* [19]. The mature miRNA is then loaded into the RNA-induced silencing complex (RISC) and regulates gene expression. The regulation of miRNA is mainly based on the principle that the seed region of the miRNA mature sequence near-perfectly matches the sequence of the target gene mRNA [24]. RISC recognizes the target region and combines it with the target region mediated by the Argonaute (AGO) protein to change the expression of the target gene and thus affect the physiological process of plants. The regulation of miRNA on target genes in plants is mainly through two modes, including target cleavage and/or translation inhibition [25]. Moreover, different miRNAs may interact with the same targets and one miRNA may regulate different targets.

miRNAs have been shown to be involved in plant growth and development through various signaling pathways, indicating that these miRNAs can function as developmental signaling molecules in plants [26]. Studies have reported that the inhibition of DCL1 and the HASTY expression of important proteins in the process of miRNA biosynthesis in plants reduces the abundance of miRNA expression, seriously affecting morphology and resulting in changes in the leaf shape and flower shape, pollination obstruction, fertility reduction, etc. [27,28,29]. miR160 negatively regulates ARF10 to maintain the homeostasis of ARF10-mediated interactions between auxin and the ABA pathways during seed germination and postembryonic development [30]. In *Arabidopsis*, miR395c negatively regulates seed germination under high salinity or dehydration stress; miR395e contain only single nucleotide differences from miR395c. However, miR395c and miR395e act as positive or negative regulators of seed germination under stress conditions [31]. miRNA controls leaf development by regulating the expression of HD-ZIP transcription factors [32]. As important members of the HD-ZIP transcription factor family, including *PHB*, *PHV*, and *REV* [33], miR165 can regulate leaf development by controlling the expression of these three target genes [34,35,36]. In rice, miR167 negatively regulates the expression of several auxin response factor genes (*ARF8* and *ARF6*) and further affects the expression of the IAA-binding enzyme gene *OSGH3-2*, which participates in the regulation of exogenous auxin and determines the content of beneficial intracellular auxin [37]. In *Arabidopsis*, miR396 inhibits cell proliferation during leaf development by inhibiting the expression of its target gene *GRF* and cell cycle-related genes [38]. The *TCP* gene of *Arabidopsis* is the target gene of miR159. The *TCP* genes in several plant species have miRNA binding sites, indicating that the miRNA-mediated regulation of leaf morphogenesis has a conserved role in plants with different leaf shapes [39]. In addition, miR156 and miR172 play a key role in the process of vegetative leaf development at the late germination stage and play an important role in the transition of plants to the growth stage [40]. Studies in maize have found that miR172 negatively regulates the number of leaves in maize during vegetative growth by controlling the expression of *glossy15* (*GL15*) [41]. The ectopic expression of apple Md-miR156h in *Arabidopsis* inhibits the expression of SPL family members *SPL17* and *SPL19*, thereby delaying plant flowering, indicating that miR156 mediates a conserved post-transcriptional regulatory pathway in apple and *Arabidopsis* [42]. miR164c can also negatively regulate the expression of transcription factors CUC1 and CUC2, increasing the petals of *Arabidopsis*. At the same time, it was found that similar members of the same miRNA family targeting the same group of genes play different functions due to different expression patterns during development [43], e.g., miR172 promotes flowering and destroys floral organ characteristics by down-regulating the expression of the target gene *APETALA2* (*AP2*) [44]. In rice, studies have shown that the overexpression of miR172 can cause spikelet deletion and floral organ deformity and that miR172b plays a role in floret development by regulating the expression of the target gene *APETALA2*-like [45]. The miR172 repression of *EAT3* (*TOE3*) is essential for floral organogenesis in *Arabidopsis*. In addition, SPL3 targeted by miR156 can directly activate the expression of *TOE3*, indicating a novel signal interaction between miR156 and miR172 in the process of flower organ formation [46]. Leaf senescence is controlled by the age of plant development and is aggravated by environmental stresses such as drought, high temperature, and salinity [47]. Overexpressing SlymiR208 in tomatoes significantly induced the early leaf senescence phenotype in *SlIPT4* gene-silencing transgenic plants, indicating that SlymiR208 positively regulates leaf senescence in tomato mainly by regulating *SlIPT2* and *SlIPT4,* which are related to cytokinin synthesis [48]. ORESARA1 (ORE1) is a key senescence regulator in *Arabidopsis thaliana*, and miR164 is involved in the regulation of leaf senescence by inhibiting *ORE1* gene expression at the post-transcriptional level [49,50]. These findings indicate that miRNAs play an important role in plant development, participating in the regulation of seed germination, stem, leaf, flower, and other different organ development.

miRNA-mediated post-transcriptional regulation has been shown to be involved in plant responses to a variety of abiotic stresses [51]. To identify miRNAs and their target genes under drought stress in peach and almond trees, qPCR was used to analyze the expression levels of miR156, miR159, miR160, miR167, and miR171 under moderate and severe water shortage conditions [52]. miR166 can improve the drought resistance of rice by causing morphological changes such as leaf curl and xylem diameter reduction [53]. The lateral root growth of transgenic rice seedlings overexpressing TIR1 and AFB2 resistant to miR393-cleaved forms was no longer inhibited by ABA or osmotic stress. This indicates that the miR393-mediated attenuation of the auxin signal can regulate the adaptation of plant roots to drought stress [54]. In addition, the overexpression of OsmiR393 and OsmiR393b in rice could improve the sensitivity of transgenic rice to salt stress, and the overexpression of OsmiR393 in *Arabidopsis* leads to the same phenotype [55]. Wheat TaMIR1119 plays an important role in regulating plant drought tolerance by regulating plant osmotic accumulation and photosynthesis and improving ROS homeostasis in cells [56]. The highly conserved miR156/SPL module plays an important role in balancing plant growth and the stress response. In *Tamarix chinensis*, the miR156/SPL module plays a regulatory function in mediating the response to salt stress [57]. miRNA is also involved in the regulation of the plant response to extreme environmental temperatures. In sunflower, miR396 responds to heat stress by regulating the expression of the target gene *HaWRKY6* [58]. In *Arabidopsis*, low temperatures can induce the up-regulation of miR393 and miR319c [59]. The overexpression of miR397a can affect the expression level of the *COR* gene downstream of the cold tolerance gene *CBF*, improving the tolerance of transgenic plants to low temperatures [60]. As a key factor of cold stress induction, miR319 is induced by cold stress in a variety of plants. The response of 12 miRNAs in sugarcane to cold stress identified the differentially expressed miR319 under normal conditions and low-temperature stress [61]. Subsequently, 18 cold-responsive miRNAs were identified using microarray in rice, and most of them were found to be down-regulated by cold [62]. Overexpressing OsmiR319b increased the proline content and survival rate, and significantly increased resistance to low temperatures [63]. It has also been found that the expression levels of SlymiR166 and SlymiR319 in tomato were increased under cold stress conditions [64]. Other abiotic stresses, including oxidative stress and nutrient stress such as nitrogen and phosphorus deficiency, also seriously restrict plant growth. A total of 144 miRNAs related to hydrogen peroxide (H_2_O_2_) stress were identified by next-generation sequencing technology combined with qPCR and 5′ RACE analysis in *Brachypodium distachyon*, and their target genes were analyzed, revealing the response and defense mechanism to oxidative stress at the post-transcriptional regulatory level [65]. In addition, the phosphoric acid transporter *NtPT2* gene was up-regulated in TamiR408 overexpressing plants wherein the overexpression of TamiR408 showed stronger stress tolerance, higher biomass, and photosynthate under low phosphorus conditions [66]. Finally, the expression level of *Arabidopsis* miR167a is significantly increased under low nitrogen stress, which can affect the lateral root growth under low nitrogen stress by targeting *ARF6* and *ARF8* [67].

Comparable to abiotic stress, biotic stress, including viruses, bacteria, fungi, insect pests, and nematode parasites, also affects the growth and development of plants [68]. miRNAs have been identified to be involved in the regulation of biotic stress and the immune response in plants. There are many common diseases in plants, and different plants are infected with different diseases. In *Arabidopsis*, A total of 293 known miRNAs and 6 potential novel sRNAs were identified from 15 small RNA libraries in post-inoculation leaves with *Phytophthora capsici* (*P. capsici*) using high-throughput sequencing [69]. miR38-3P, a novel miRNA, was highly induced in expression after infection of the pathogen *Sclerotinia sclerotiorum,* which might target AT3G03820 in the involvement of *Arabidopsis*-*Sclerotinia* interaction [70]. To enhance the resistance ability of *Arabidopsis* against pathogen infection, a Bacillus velezensis FZB42-treated library and control library were constructed, and 11 known miRNAs and 4 novel miRNAs were differentially expressed after FZB42 inoculation [71]. These results showed that miRNAs and their targets have closely associated with defense response. In wheat, small RNA high-throughput sequencing was used to screen and identify miRNAs involved in powdery mildew stress response. The results showed that 24 miRNAs might be involved in the powdery mildew stress response, among which, 8 miRNAs responded to powdery mildew infection in susceptible wheat cultivar Jingdong8 (JD8). miR2001, miR2006 and miR2011 were down-regulated after powdery mildew infection, and miR393, miR444, miR827, miR2005, and miR2013 were up-regulated. A total of 3 miRNAs responded to powdery mildew infection in JD8-*Pm30*, a near-isogenic resistant line of JD8, including miR171 down-regulated and miR2008 and miR2012 up-regulated after powdery mildew infection. There were 10 miRNAs that responded to powdery mildew infection in both JD8 and JD8-*Pm30*, among which miR156, miR159, miR164, and miR396 were down-regulated after powdery mildew infection [72]. In tomato, a total of 79 plant miRNAs and 40 potential candidate miRNAs were differentially expressed after *Cucumber mosaic virus* (CMV)-infection [73]. The fungus *Magnaporthe oryzae* (*M. oryzae*) is the most important disease in rice; the expression level of rice miR319 was induced by *M. oryzae* strain Guy11. miR319 and its target gene *TEOSINTE BRANCHED/CYCLOIDEA/PROLIFERATING CELL FACTOR1* (*OsTCP21*) may participate in the suppression of *M. oryzae* infection [74]. In addition, a previous study showed that suppressing the expression of miR482 and increasing the level of NBS (nucleotide-binding site)-LRR (leucine-rich repeat) transcript could increase the resistance of cotton to *Verticillium dahliae* [75]. miR482 and its target genes NBS-LRR are involved in regulating potato resistance against *Verticillium dahliae* infection in potato [76]. Moreover, miR472a could also target NBS-LRRs and is involved in the effective defense against the necrotrophic fungus *Cytospora chrysosperma* in poplar [77].

The miRNAs induced under various stresses can fine-tune the expression of target genes that function in the regulation of stress tolerance in *B. napus*. Hence, it is necessary to understand miRNA regulation during combat stress conditions. In the present review, we discuss miRNA regulation in plant development and biotic and abiotic stress responses in *B. napus* from recent research progress, dissected functional studies to decipher the regulation network behind miRNA-based stress tolerance, and designed stress-resilient rapeseed through the manipulation of miRNAs.

## 2. MiRNAs and Development Regulation in *B. napus*

miRNAs have been investigated for the regulation of plant development in diverse plant species, for instance, *Arabidopsis* [39], rice [78], wheat [79], tomato [80], maize [81], strawberry [82], sugarcane [83], apple [84], sweet potato [85], and ornamental gloxinia [86]. With the rapid development of biotechnology, such as high-throughput sequencing, thousands of miRNAs also were identified under rapeseed development [87]. As shown in Table 1, the known miRNAs in *Arabidopsis* and rice were used to search for potential miRNAs in the EST and GSS databases of *B. napus* [88]. After strict filtering criteria, 21 miRNAs were detected, and 67 potential target genes were further found through a search of the mRNA database [89]. The branch angle determines the planting density of *B. napus* in the field, and a smaller branch angle can increase the planting density of *B. napus*, thus improving the yield of *B. napus*. Sequences of two *B. napus* varieties with different branch angles reveal the relationship between miRNA-related target genes and auxin or BR signaling pathways, which can be finely regulated by changing the expression of these genes in *B. napus* [90,91]. The 17 *euAP2* genes targeted by miR172 were identified and these genes showed high expression in the floral organs in *B. napus*, suggesting that miR172-*euAP2* may function in flower development [92]. Recently, 12 small RNA libraries of genic male sterility lines in rapeseed were constructed and sequenced to analyze the differential expression of miRNAs in regulating pollen development, the results showed that miR159 may regulate the fertility in rapeseed [93]. Meanwhile, silique and seed development are also important points to improve the production and quality of rapeseed [94]. Rapeseed genotypes with long and short siliques were used to establish small RNA libraries and 17 differential expressed miRNAs were identified. These miRNAs, such as miR159, miR319, miR160, miR399, miR408, miR827, and miR2111, may be involved in cell proliferation, auxin signal transduction, and inorganic phosphate/copper deficiency to control silique development [95]. Some miRNAs, such as miR159, miR6029, and miR827, were identified to regulate the thickness of the pod canopy for yield information [96]. Moreover, more than 500 miRNAs were identified during seed maturation from 10–50 days after flowering in rapeseed using next-generation sequencing; among them, miR156, miR159, miR172, miR167, miR158, and miR166 were found to be involved in the regulation of seed development and maturation [97]. The composition and content of fatty acids affect the quality of rapeseed oil [98,99]. Computational studies using high-oil-content and low-oil-content rapeseed cultivars identified some miRNAs that may be involved in regulating the oil content of *B. napus* [100]. Other studies have also shown that miRNAs play a role in the synthesis of fatty acids, and miRNAs participate in the formation of acetyl-CoA and carbon chain desaturase, regulating the level of long-chain fatty acids, *β*-oxidation, and lipid transport and metabolism, thereby affecting the synthesis of fatty acids in *B. napus* [101,102]. Therefore, the miRNA regulation of silique development and fatty acid synthesis may have a role in the yield of *B. napus*, possibly influencing oil content.

## 3. MiRNAs and Abiotic Stress in *B. napus*

Abiotic stress is the most widely studied miRNA-mediated regulation in the plant, including drought stress [103], salt stress [104], cold stress [105], cadmium stress [106], and nutrient deprivation [107]. Drought and salt stress severely affect the germination of rapeseed [108]. To investigate the regulatory function of miRNAs in the germination of rapeseed under drought and salt stress (Table 2), the rapeseed seeds were exposed to a drought and salt treatment, and then the 85 known miRNAs and 882 novel miRNAs were identified by high-throughput sequencing. Among them, miR156, miR169, miR860, miR399, miR171, and miR395 were down-regulated and miR172 was up-regulated under drought or salt stress [109]. Further, repressing the expression of miR169 improved drought resistance by targeting *NF-YA8* in *B. napus* [110]. Other than drought and salt stress, cold stress has also been studied in rapeseed; a total of 70 known miRNAs and 126 novel miRNAs were identified in leaf tissues under 4 °C conditions, and 25 known and 104 novel miRNAs were differentially expressed in rapeseed [111].

Cadmium (Cd) is one of the most toxic heavy metals and with its high mobility in soil, it is easily absorbed and accumulated in plants [112,113]. Excessive accumulation of cadmium in plants will affect plant development and cell function, and sometimes have a fatal impact on plants [114]. A total of 84 miRNAs were identified from four small RNA libraries and 802 targets were identified for 37 miRNA families by Cd-treated rapeseed [115]. *BnNRAMP1b* is regulated by miR167 in rapeseed at the post-transcriptional level. *BnNRAMP1b* is related to the transportation of intracellular and extracellular environmental substances in *B. napus*, which can help heavy metal Cd into the rapeseed cell and lead to cell poisoning. The negative regulation of miR167 on *BnNRAMP1b* can effectively inhibit this process and help rapeseed nullify Cd damage [116]. miR395 and miR158 were also confirmed to play a role in the Cd detoxification of *B. napus* [117,118]; overexpression of miR395 increased Cd tolerance in *B. napus* [117].

Rapeseed growth and seed production need optimal nutrient allocation under sub-optimal conditions [119]. Many miRNAs have been identified and characterized from the phloem in rapeseed [120]. A previous study showed that miR399 was induced by phosphate (P) starvation, and miR399 is potentially involved in long-distance communication via the phloem following phosphate deprivation [121]. miR398 and miR395 were up-graded in phloem sap under copper and sulfate starvation respectively [120]. In addition, miRNA microarray results showed that miR395 is also a potential long-distance molecule for transporting via the phloem [122]; a miR2111, miR169, and miR827-like sequence can respond to P and nitrogen (N) status in rapeseed phloem sap [123]. Furthermore, degradome sequencing and RT-qPCR assays revealed that miR827 regulates the process of N-induced leaf senescence, and rapeseed root development under N deficiency depends on the regulation of the miR171-*SCL6* and miR160-*ARF17* pathways in rapeseed [124]. Taken together, such miRNAs were found to be involved in the regulation of abiotic stress, but little is known about the impact of stress-related miRNAs on their target genes in *B. napus*. Therefore, miRNAs and targets can become the new targets for designing abiotic stress-resilient rapeseed.

**Table 2:** The functions of miRNAs in *B. napus* under biotic and abiotic stresses.

Stress	MicroRNAs	References
Salt and drought stress	Multiple miRNAs	[109,111]
Drought stress	miR169	[110]
Cadmium stress	miR158, miR167, miR395, etc.	[112,113,114,115,116,117,118]
Nutrient stress	miR395, miR398, miR399, etc.	[120,121,122,123]
Vascular disease	miR168	[125]
Sclerotinia rot	miR159, miR5139, miR390, etc.	[126,127,128]
Clubroot disease	Multiple miRNAs	[129]

## 4. MiRNAs and Biotic Stress in *B. napus*

Pathogen invasion, bacteria, and insects are the most common biotic stresses. However, vascular disease and sclerotinia rot are the most destructive diseases in Brassica species, causing significant crop losses every year [130,131] (Table 2). The fungi spread in the plants by means of hyphal growth or conidia transporting from infected root to shoot [125]. miR393 was the first miRNA-regulated plant antibacterial *PTI* (pattern-triggered immunity) through the auxin signaling pathway in *Arabidopsis* [132]. In *B. napus*, vascular disease is caused by *Verticillium longisporum* (*V. longisporum*). A total of 893 *B. napus* miRNAs, including 360 conserved and 533 novel miRNAs, were identified from *V. longisporum* infected/noninfected roots, and miRNA168-AGO1 was found to be associated with the compatible plant and *V. longisporum* interaction [125]. Some miRNAs responsive to *Sclerotinia sclerotiorum* (*S. sclerotiorum*) infection have been identified by high-throughput deep sequencing, and their targets were predicted using degradome sequencing to explain the complex mechanism of *S. sclerotiorum* infection [126,127]. In addition, the expression of miR159, miR5139, and miR390 altered in response to *S. sclerotiorum*. A miR1885-triggered disease resistance gene-derived secondary sRNA locus was also identified and verified with degradome sequencing [128]. On top of that, the differential expression of miRNAs was identified in the potential regulation of clubroot disease with *Plasmodiophora brassicae* [129]. Overall, in the process of pathogen infection, the specific functional role of miRNA in the defense response of fungi needs to be studied further.

## 5. Discussion and Future Perspectives

*Brassica napus* has a large and complex genome due to the hybridization between *Brassica rapa* and *Brassica oleracea* [133]. A variety of natural disasters limited the growth and development of rapeseed to a great extent, affected the yield and quality of rapeseed, and endangered the food safety of China. In the face of the different stresses, developing stress-tolerant rapeseed is one of the most economical and effective methods for biological breeding. With the release of the *B. napus* genome and the wide application of high-throughput sequencing technology, the research on *B. napus* molecular breeding has entered an explosive stage. At present, the psRNATarget database [134] and degradome sequencing [135] are powerful tools to predict and validate the target genes of known miRNAs, illuminating the regulatory network of miRNAs and their target genes in the normal development and response to the rapeseed’s detrimental environment. Although many miRNAs have been identified based on the next-generation sequencing in rapeseed under different stresses, little is known about the molecular basis of miRNAs in *B. napus*. Therefore, after the identification of rapeseed miRNA under different stresses, further studies should be focused on the exploration of function, which is essential to develop stress-tolerant improvement through miRNA manipulation. Alternatively, miRNAs may affect rapeseed development and stress tolerance through various auxin pathways, therefore, the crosstalk of miRNAs and plant hormones should be verified to expand our knowledge of the role in rapeseed miRNA function; their associated regulatory networks represent a compelling area of research to pursue in the future. Another aspect we should focus on is that one miRNA may have multiple targets, which would cause different effects on rapeseed growth, development, and stress tolerance. Whether there are common characteristics and functions of the same miRNAs in the regulation of different stresses in rapeseed, and how miRNAs help rapeseed resist stress by regulating target genes, still need to be confirmed by more studies. We should also consider how to select optimized targets and balance between normal development and improving the tolerance of different stresses to develop the ideal rapeseed for high resistance and high yield.

There are important directions to take regarding the interaction between rapeseed and microorganisms for improving yield and stress tolerance. The subterranean microbiota of plants plays a crucial role in plant growth and health, as root-associated microbes can perform important ecological functions. *S. sclerotiorum* is a pathogenic bacterium that widely infects the reproductive growth of rapeseed and causes the loss of rapeseed production. A previous study has identified more miRNAs in response to *S. sclerotiorum* infection by high-throughput deep sequencing. However, its in-depth analysis to untangle the complex regulatory networks and their cross-talks require further research. Apart from pathogenic bacteria, non-pathogenic bacteria are inescapable functions in plants. For example, the rhizobial endophytes have the ability of nitrogen fixation to promote soybean growth and soybean yield and improve the tolerance of abiotic stress in soybean [136,137]. In rice, plant growth-promoting bacteria (PGPB) are not only effective in improving rice productivity but also in combating bacterial rice pathogens. Applications of PGPB provide an eco-friendly alternative to agroecosystems [138]. In addition, the root bacteria of *B. napus* were found to enhance the rapeseed yield [139], whether those root bacteria may help rapeseed combat the various stresses needs to be explored. Meanwhile, we should consider the relationship between miRNA and non-pathogenic bacteria to provide new insight into their cross-talk, filling the gap in the research on the relationship between miRNA and non-pathogenic bacteria in *B. napus*.

Currently, gene transformation technologies have been applied to confer stress resilience and a high-yield capacity in plants, including *B. napus* [140]. In recent research, the stress-resilience of transgenic plants could be increased by overexpressing miRNA. Meanwhile, the inhibition of miRNA activity by target mimicry (MIM) and short tandem target mimic (STTM) technology has been applied in various plants [141,142]. MIM technology, with the non-protein coding gene *IPS1*, contains a motif with sequence complementarity to the miRNA, resulting in the repression of miRNA cleavage [143]. STTM technology can be used as an updated version of MIM for enhancing the inhibition of miRNA activity [144]. In rapeseed, the repression of miR169 by target mimicry can impart tolerance to drought stress [110], but STTM technology has not been used in rapeseed. CRISPR/Cas9 technology is now a very popular method for genome editing. Currently, CRISPR/Cas9 can mutate the miRNA genes to uncover their function [145] or edit the miRNA recognition sites of target genes to change their expression [146]. Regarding rapeseed, a few studies have utilized the CRISPR/Cas9 system to edit genes associated with plant development [147,148], pod shattering [149,150], seed production [151], fatty acid composition [152], and responses to various stresses [153], however, gene editing of miRNAs has not been used in rapeseed, and there are more prospects to develop and use a variety of tools for miRNA manipulation in *B. napus*. Sometimes, the role of different miRNAs is also a non-negligible relationship, which is worth our consideration and in-depth exploration. In summary, the identification of stress-resistance-related miRNAs and construction of the plant miRNA network, as related to development or stress resistance, can lead to improved plant defenses and yield through miRNA manipulation in *B. napus*.

## 6. Conclusions

With the continuous progress of biotechnology and the reduction in technology costs, the research on miRNA prediction and regulation is becoming increasingly extensive in plants. In *B. napus*, the vast amount of transcriptomic data on development and biotic and abiotic stress offer one possibility to exploit the regulatory network of miRNA-mRNA. The advances in tools and the sequence information from related plants also provide a reference for more unknown miRNAs in rapeseed to learn more about the characteristics of miRNAs for the improvement of favorable traits in *B. napus* and can provide the basis for the breeding of multiple-resistant rapeseed.

## Figures and Tables

**Table 1:** The functions of miRNAs in *B. napus* development.

Functions	MicroRNAs	References
Branch angle regulation	Multiple miRNAs	[90,91]
Flower development	miR172	[92]
Male sterility	miR159	[93]
Silique development	miR160, miR2111, miR399, miR827, and miR408	[95]
Thickness of pod canopy	miR159, miR6029, and miR827	[96]
Seed development	Multiple miRNAs	[94,97,100]
Fatty acid and content	Multiple miRNAs	[98,99,101,102]

## Data Availability

Not applicable.
